# A Staghorn Calcium Phosphate Stone in a Child With Sanjad-Sakati Syndrome: An Iatrogenic Manifestation?

**DOI:** 10.7759/cureus.23032

**Published:** 2022-03-10

**Authors:** Mohammad A Alomar, Mohammad A Alghafees, Raouf M Seyam, Abdulaziz S Aljurayyad, Reema S Aldhalaan, Khalid M Alshuwaier, Yasser M Alkharashi, Abdulrahman L Albassam

**Affiliations:** 1 Urology, King Faisal Specialist Hospital and Research Centre, Riyadh, SAU; 2 College of Medicine, King Saud Bin Abdulaziz University for Health Sciences, Riyadh, SAU; 3 Urology, King Saud University Medical City, Riyadh, SAU; 4 Urology, King Fahad Specialist Hospital, Buraydah, SAU; 5 College of Medicine, Alfaisal University, Riyadh, SAU

**Keywords:** pediatric rare diseases, iatrogenic stones, saudi arabia, kidney stones, sanjad-sakati syndrome

## Abstract

Sanjad-Sakati syndrome (SSS) is an autosomal recessive genetic condition, with the first report discussing this condition presented in Saudi Arabia. This case report describes an iatrogenic stone as a result of hypocalcemia overtreatment, along with its subsequent management procedure. The current literature concerning the iatrogenic stone occurrence and the operative outcome of percutaneous nephrolithotomy in individuals with SS is scarce, warranting further investigation.

## Introduction

Sanjad-Sakati syndrome (SSS) is an autosomal recessive genetic disease, with the first report discussing this condition presented in Saudi Arabia [1]. This disorder is exclusively found among Arabian individuals; however, case studies from non-Arab nations have also been recorded [2]. Hypoparathyroidism-retardation-dysmorphism syndrome is classified in the Online Mendelian Inheritance in Man® (OMIM) as SSS. On chromosome 1, at 1q42-43 loci, Parvari et al. discovered a gene. Gene mutation that codes for the *tubulin-specific chaperone E* (*TBCE*) may cause this condition [3].

SSS is characterized by severe growth retardation, typical facial features, low intelligence quotient, and congenital hypoparathyroidism. Seizures, mental and physical impairment, hyperphosphatemia, and hypocalcemic tetany are some of the symptoms that children with this condition experience. The primary management includes treating hypoparathyroidism. Therefore, calcitriol (active vitamin D) and calcium are the mainstays of treatment, and the iatrogenic overcorrection of hypocalcemia can potentially cause renal stones (iatrogenic stones). Renal stones with a diameter of >2 cm and staghorn stones are mainly treated with percutaneous nephrolithotomy (PCNL) [4]. Here, we present a case of staghorn renal stone in a pediatric patient with SSS that warranted PCNL.

## Case presentation

**Figure 1:**
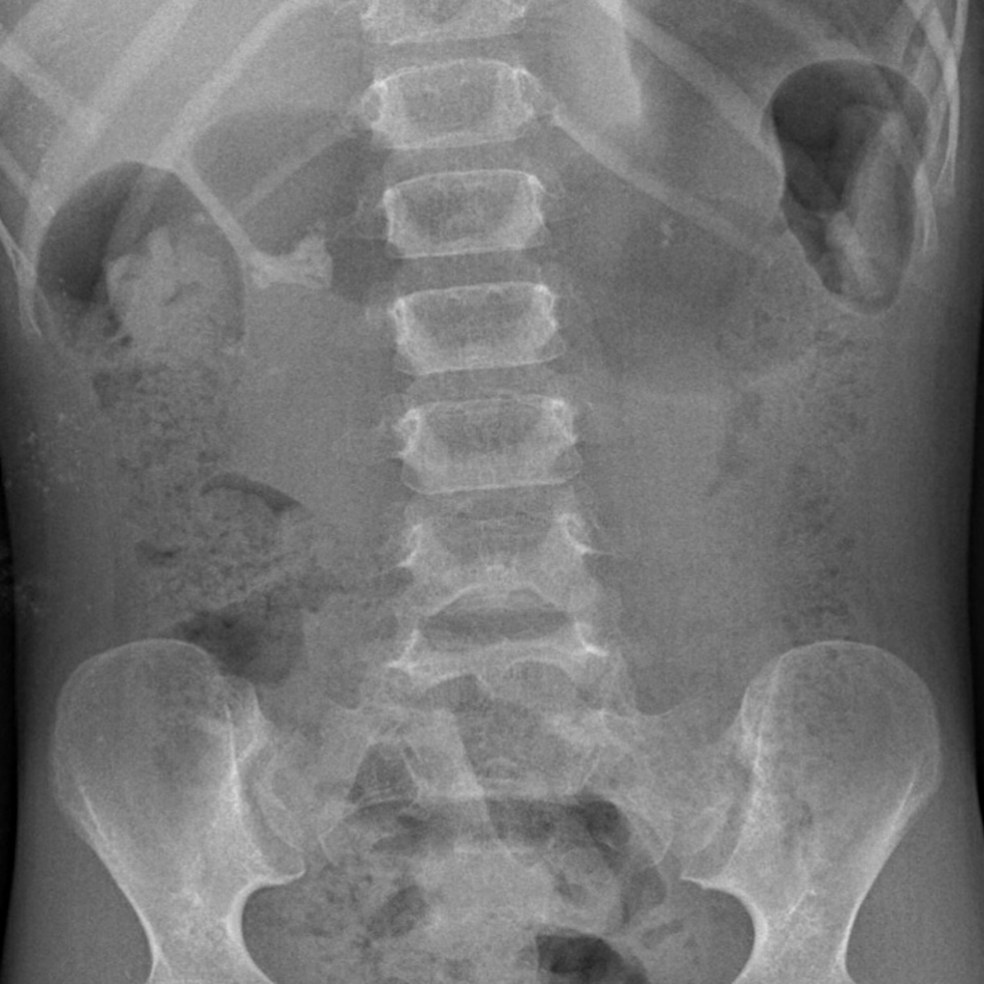
Preoperative plain X-ray image showing a partial staghorn calculus.

**Figure 2:**
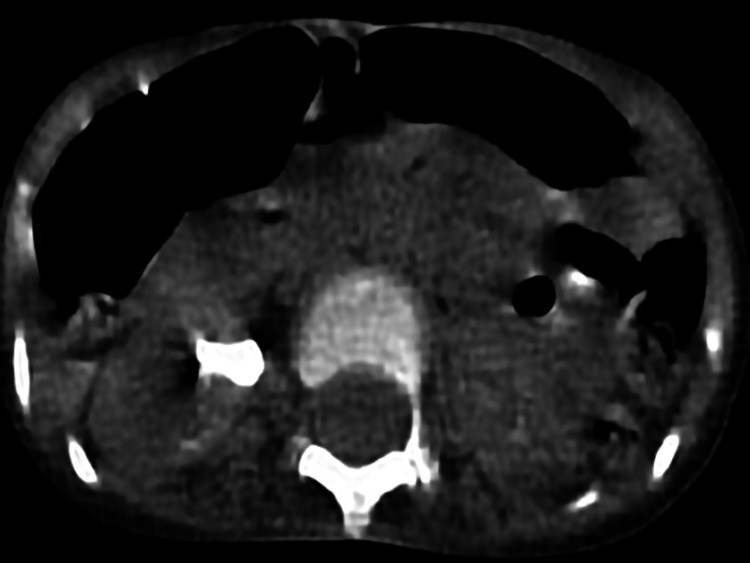
Preoperative CT scan showing a partial staghorn stone along with medullary nephrocalcinosis.

An 11-year-old female with SSS presented to the urology department with renal stones after an initial workup for hypocalcemia by her pediatrician. The patient had no relevant active or resolved medical history nor any notable family history. On physical examination, the patient was significantly underweight, with a body mass index of 16.7 kg/m^2^, and was noticeably shorter for her age, while microcephaly was present. She had experienced multiple episodes of hypocalcemia that required medical treatment. In addition, she had a history of skipping multiple follow-up visits as she lived in the countryside away from any specialized centers. She had no notable history of urinary tract infections. On imaging, X-ray and computed tomography (CT) scan showed a partial staghorn renal stone of 12 mm in diameter along with medullary nephrocalcinosis (Figures 1, 2). Hence, the patient was admitted for PCNL.

**Figure 3:**
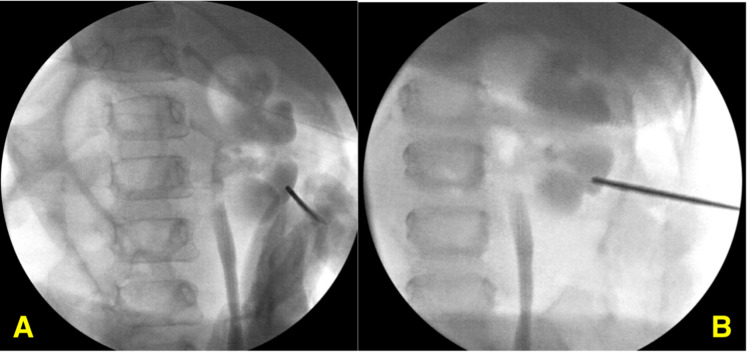
(A) Intraoperative fluoroscopy image showing initial puncture using the C-arm with 20% angulation. (B) Intraoperative fluoroscopy image showing the needle trajectory in an oblique view reaching the collecting system.

PCNL was done using the bullseye technique targeting the most posterior middle pole calyx (Figures 3A, 3B).

**Figure 4:**
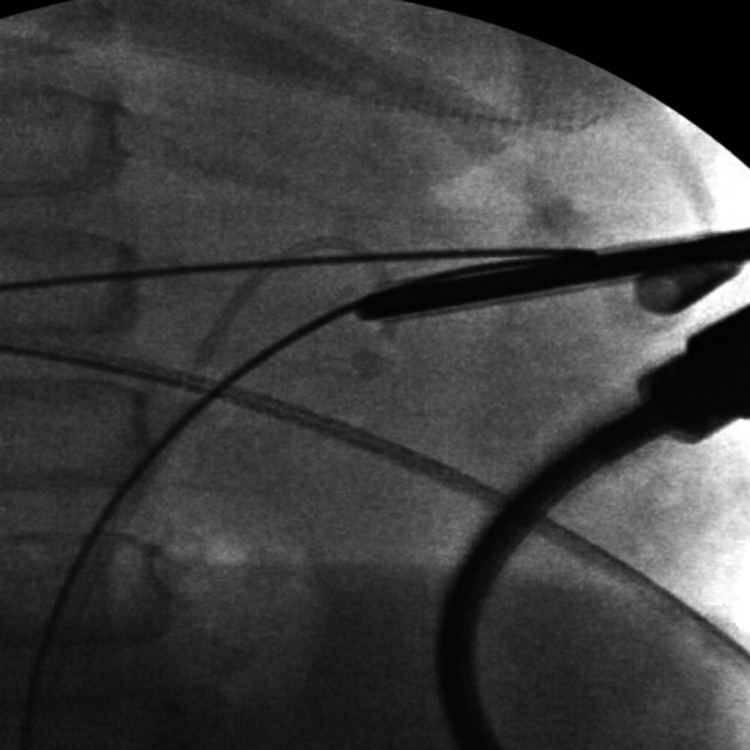
Intraoperative fluoroscopy image showing a semi-rigid ureteroscope removing small stone fragments at the end of the procedure after stent placement.

Subsequently, a hybrid hydrophilic guidewire was passed down to the bladder, dilating the skin to 10F. Subsequently, an 11F mini-PCNL access sheath was inserted over the guidewire. Initially, a 6.9F semirigid ureteroscope was used, complete fragmentation was achieved using a 200 µm Holmium laser fiber, and fragments were basketed (Figure 4).

**Figure 5:**
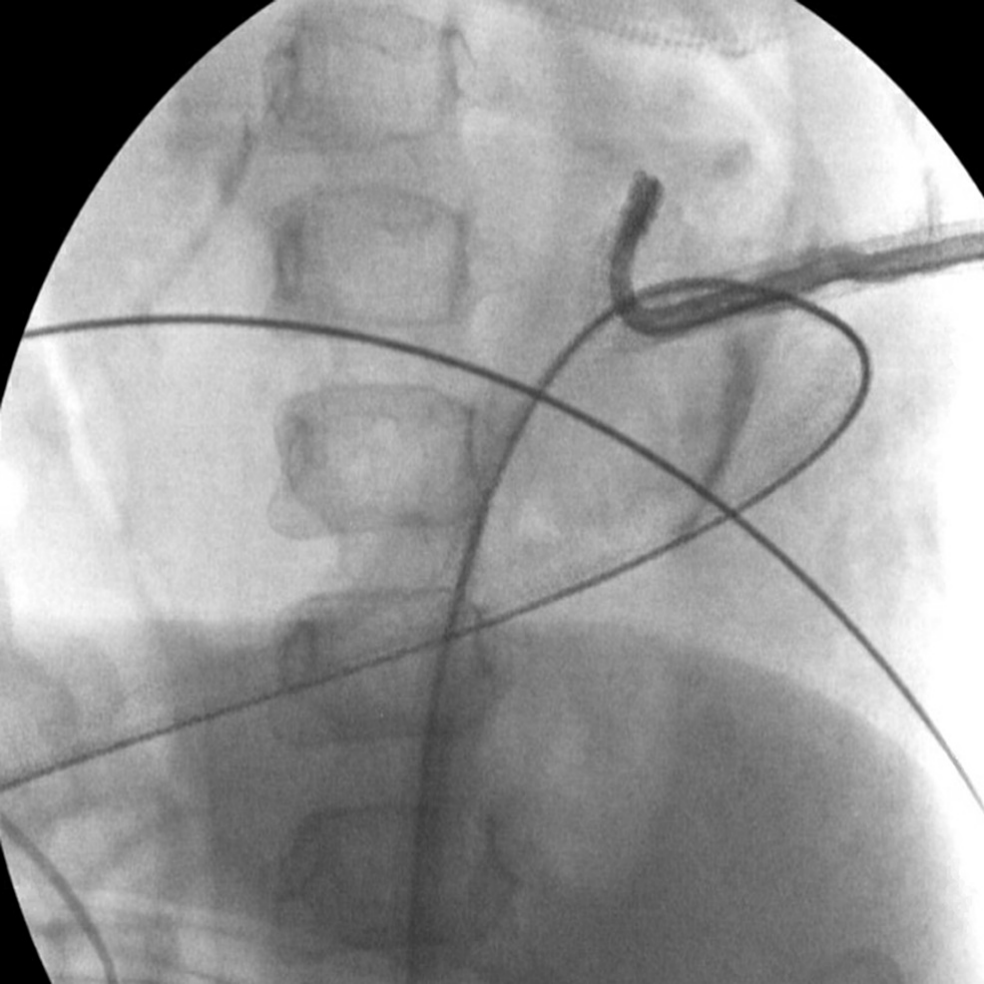
Intraoperative fluoroscopy image showing a flexible nephroscope checking the collecting system for remnant stones.

Afterward, a 7.5F flexible ureteroscope was used to check the rest of the collecting system (Figure 5).

Then, a 3.7F antegrade ureteral stent was inserted. Following a normal X-ray nephrostogram, we decided to proceed without inserting a nephrostomy tube. The patient was extubated uneventfully. After one month, the patient was readmitted to remove the stent with complete stone resolution apart from the previously seen parenchymal stone. Stone analysis showed 100% calcium phosphate by infrared spectroscopy. She had an uneventful postoperative course and was discharged following one month of admission and careful monitoring.

## Discussion

SSS is an autosomal recessive condition initially defined in 1988 and 1991 by Sanjad et al. in Saudi Arabia [5,6], along with reports from some other countries [2-7]. Parvari et al. utilized linkage disequilibrium and homozygosity to find the gene on chromosome 1q42-43 in 1998 [3]. According to the research published in 2002, autosomal recessive Kenny-Caffey and SSS are caused by *TBCE* mutations [8].

This case report describes an iatrogenic staghorn stone caused by hypocalcemia overcorrection in SSS. SSS has a wide range of endocrinological symptoms. According to Bashar et al., hypoparathyroidism remains the most common manifestation of this condition [9]. This leads to high phosphorus and low blood calcium levels. The iatrogenic overcorrection of hypocalcemia can cause renal stones [10]. Arlt et al. conducted a cross-sectional study to analyze calcium homeostasis. The study aimed to analyze calcium homeostasis in 25 PO-hypoparathyroidism patients on conventional therapy. The study showed 8% of the patients had nephrolithiasis [11]. Furthermore, a retrospective study included 8,051 patients with hypoparathyroidism and 40,466 individuals in the control group. The study found a significantly increased risk of nephrocalcinosis in patients with hypoparathyroidism in comparison to those without hypoparathyroidism during five years of follow-up. Nevertheless, the underlying etiology behind this phenomenon remains uncertain. However, one theory suggests that conventional therapy for hypoparathyroidism is the underlying cause. The theory hypothesized that the pathophysiology is potentially through hypercalciuria. Alternatively, another hypothesis suggests the course of the disease and scarcity of normal physiological effect of parathyroid hormone on renal tubules being a risk factor for nephrocalcinosis and nephrolithiasis in hypoparathyroidism [12]. Inclusively, the calcium correction in hypoparathyroidism is linked with nephrolithiasis and many other renal complications [13]. In our case, the confirmation of calcium phosphate stone on stone analysis further solidified the fact that it was iatrogenic. To our knowledge, this is the second case of a renal stone in an SSS patient and the first discussion of its management with PCNL [14]. A better acknowledgment of this complication is essential for early diagnosis and improved disease management.

This case has some limitations necessitating future investigations. First, because the patient lived in the countryside, she missed several follow-up visits with her specialized pediatrician. Consequently, we could not get lab values to identify the etiology of the stone with complete certainty. However, the multiple hypocalcemia episodes which required vigorous treatment, the lack of urinary tract infection history, the lack of follow-up to modify treatment, and the size of the stone support our theory that the stone was iatrogenic.

## Conclusions

This report describes an iatrogenic staghorn stone and its management procedure. To our knowledge, this is the second report of a renal stone in an SSS patient and the first discussing its management in detail. Given the fact that SSS patients experience recurrent severe hypocalcemia episodes that require vigorous treatment, more investigation is needed on such iatrogenic complications. This would allow the optimization of prevention and management to ensure the highest quality possible for the short life that these patients live. Moreover, formulating a regional registry for patients with such a rare condition is recommended to exchange experience and perform larger-scale studies. Moreover, as evident by the lack of monitoring in our patient, more light needs to be shed on the lack of specialized care in rural areas for children with genetic diseases. This is especially important because consanguinity is more common in rural communities leading to a higher prevalence of autosomal recessive disorder patients, such as SSS, in these areas.
